# Case Report: Familial hCG syndrome with elevated hCG level concurrently in blood and cerebrospinal fluid

**DOI:** 10.3389/fendo.2026.1806387

**Published:** 2026-03-26

**Authors:** Danyi Wang, Mengtian Huang, Jun Zhang, Rujiang Zheng, Qiuli Chen, Song Guo, Bing Wang, Yanhong Li, Huamei Ma

**Affiliations:** 1 Department of Pediatrics, The First Affiliated Hospital, Sun Yat-sen University, Guangzhou, China; 2 Department of Pediatrics, Peking University Shenzhen Hospital, Shenzhen, China

**Keywords:** blood, case report, cerebrospinal fluid, chorionic gonadotropin, family

## Abstract

**Objective:**

To describe a rare pediatric case of familial human chorionic gonadotropin (hCG) syndrome presenting with concurrent elevation of beta-hCG (β-hCG) in both blood and cerebrospinal fluid (CSF). This report aims to expand the phenotypic spectrum of this condition and discuss diagnostic challenges to avoid misdiagnosing this benign disorder as an intracranial malignancy.

**Methods:**

Clinical data were collected from the proband, an 8-year-9-month-old girl presenting with central precocious puberty (CPP) and unexplained hCG elevation. To evaluate the differential recognition of hCG variants by diverse detection antibodies, serum and CSF β-hCG levels were cross-monitored using both Abbott Architect and Roche Elecsys platforms. Additional evaluations included magnetic resonance imaging (MRI), computed tomography (CT), and pathological examination. Whole-exome sequencing (WES) and family screening of first-degree relatives were conducted to identify the etiology. A literature review regarding familial hCG syndrome was also conducted.

**Results:**

The patient was initially diagnosed with CPP due to breast development and accelerated growth. During routine screening to exclude tumor-associated precocious puberty, she was found to have elevated serum β-hCG (132.1–136.3 IU/L, Roche Elecsys) and was referred to our hospital. Imaging revealed a pineal cyst without evidence of tumor. Both serum and CSF β-hCG levels were elevated (45.58 and 103.22 IU/L, respectively; Abbott Architect), with CSF levels exceeding serum. Despite chemotherapy, radiotherapy, and pineal cyst resection (histology: central neurocytoma), hCG remained persistently elevated. Given the benign clinical course and lack of therapeutic response, segregation analysis was subsequently performed. Elevated serum β-hCG levels were identified in the patient’s asymptomatic mother and prepubertal younger brother (43.79 and 64.58 mIU/mL, respectively; Abbott). Whole-exome sequencing was negative. Familial hCG syndrome was finally diagnosed, and it was determined that her CPP was an independent, concurrent condition. Oncological treatments were ceased, and the girl continued gonadotropin-releasing hormone agonist (GnRHa) treatment.

**Conclusion:**

This case report describes a novel clinical finding in familial hCG syndrome characterized by concurrent elevation of β-hCG in both serum and CSF. This finding significantly expands the phenotypic spectrum of this benign condition.

## 1 Introduction

Familial human chorionic gonadotropin (hCG) syndrome is a rare benign condition characterized by elevated serum or urine hCG levels in the absence of pregnancy, malignancy, or pituitary disease (1). The diagnosis is confirmed when similar manifestations are observed in family members, including siblings or parents (2). The estimated incidence is approximately 1 in 60,000, with 14 kindreds globally (10 in the United States, 1 in New Zealand, 1 in Belgium, 1 in Hong Kong, and 1 in Spain) (2–7) and one single case from Spain (8). Affected individuals typically produce atypical hCG isoforms, including free β-subunit hCG (hCGβ), C-terminal peptide (CTP)-deleted variants, or free hCGβ or hCG with a mutation that impairs CTP recognition (3), which are biologically inactive at the hCG receptor and do not impair reproductive function. Therefore, it is considered an asymptomatic benign condition. However, it is frequently misdiagnosed as a malignant tumor, leading to unnecessary treatments and surgeries, which causes significant psychological and physical stress for patients and their families. Thus, there is a need for increased clinical awareness of this syndrome. Additionally, familial hCG syndrome may exhibit autosomal dominant inheritance (2) and fluctuating hCG levels (3).

This article reports the first pediatric proband with familial hCG syndrome identified in China. Notably, this case is highly atypical in that the patient concurrently presented with an independent, separate reason for central precocious puberty (CPP) alongside familial hCG syndrome. Furthermore, this is also the first observation of elevated cerebrospinal fluid hCG levels (which exceed peripheral blood levels). This finding expands the clinical spectrum of familial hCG syndrome and underscores the need to consider this rare entity in the differential diagnosis of children with unexplained hCG elevation, in order to prevent unnecessary and potentially harmful oncologic treatments.

## 2 Case description

An 8-year 9-month-old girl was referred to our hospital on December 3, 2020 due to “breast development with accelerated growth for 1.5 years, and unexplained elevated serum hCG for over 7 months.” At age 7 years and 3 months, she had presented with bilateral breast development and accelerated linear growth. At 8 years and 2 months, evaluation revealed central precocious puberty (CPP) and markedly elevated serum β-hCG (132.1-136.3) IU/L, exceeding pediatric reference ranges (<2.39 IU/L (9) 0–3 IU/L (10), Roche Elecsys/hCG+β) with no abnormalities on cranial MRI, chest CT, or abdominal and pelvic ultrasonography. Her initial evaluation followed the 2011 Chinese guideline on precocious puberty, which recommends measuring serum β-hCG and α-fetoprotein to exclude hCG-producing germ cell tumors; this explains why β-hCG was assessed despite the absence of tumor symptoms. Baseline gonadotropin levels were within the pubertal range: LH 1.34 IU/L and FSH 4.76 IU/L. The GnRH stimulation test showed peak LH and FSH levels of 28.43 IU/L and 18.67 IU/L respectively. AFP was within normal limits (1.05 μg/L). Over the following 6+ months, blood hCG levels fluctuated between 12.7 IU/L and 20.7 IU/L (Abbott Architect,total β-hCG).

## 3 Diagnosis and treatment

**Figure 1:**
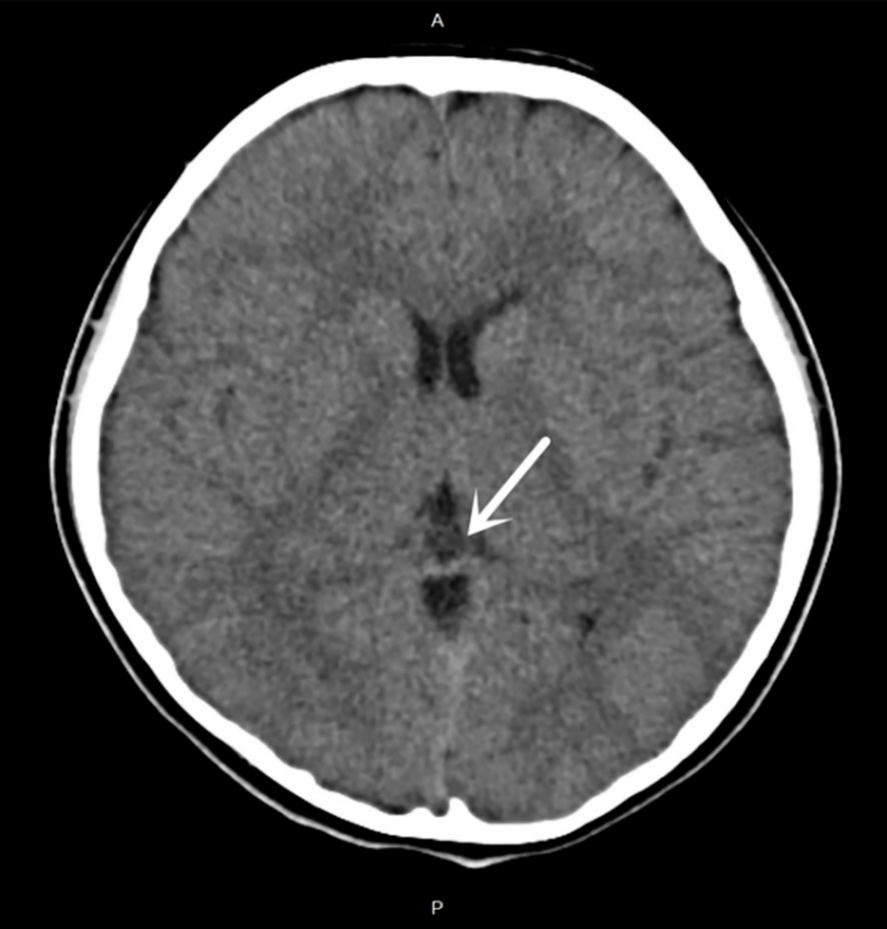
Non-contrast cranial CT showing a well-circumscribed, near-circular, water-attenuation cystic lesion located in the pineal region, consistent with a pineal cyst. White arrows indicate the pineal cyst location in axial CT image.

**Figure 2:**
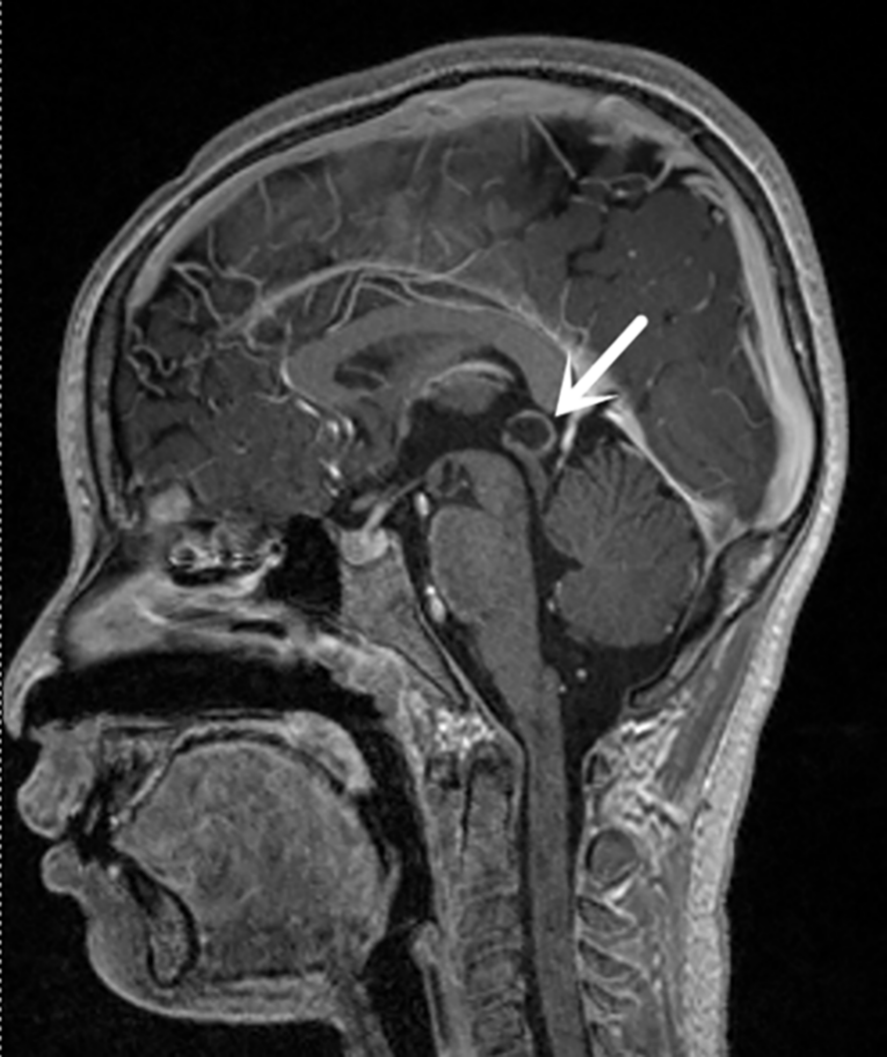
Sagittal post-contrast T1-weighted MRI demonstrating a well-defined rounded cystic lesion in the pineal region extending into the quadrigeminal cistern, consistent with a pineal cyst. White arrows indicate the pineal cyst location in sagittal MRI image.

On admission to our hospital, her height was 128.9 cm (−0.6 SD), weight 29.4 kg, and Tanner stage B3/PH1. Bone age was 11 years (Greulich–Pyle method). Laboratory examinations showed normal urinalysis and liver enzymes. The comprehensive hormonal profile, including basal and GnRH-stimulated gonadotropins, was summarized in **Table 1**. Neurological and systemic examinations were unremarkable. Serum β-hCG was elevated at 45.6 IU/L (Abbott Architect), while CSF β-hCG was even higher at 103.22 IU/L (pediatric reference range < 1.009 IU/L (11), Abbott Architect). No tumor cells were detected in the CSF. Karyotype was 46,XX. Cranial imaging revealed only a pineal cyst (**Figures 1**, **2**).

**Table 1:** Hormonal profile data of the girl at presentation.

Serum hormone (abbreviation) [unit]	Patient value	Reference range (Tanner III)
Gonadotropins & Sex Hormones Fertility hormone		
Follicle-stimulating hormone, basal (FSH) [IU/L]	6.53	0.10~7.19 (16)
Luteinizing hormone, basal (LH) [IU/L]	1.93	<7.43 (16)
Estradiol (E2) [pg/ml]	29	<86 (16)
Testosterone (T) [ng/ml]	0.23	<0.42 (16)
Prolactin (PRL) [ng/ml]	8.63	3.92~23.11(1); 5~25 (17)
Progesterone (P) [ng/ml]	0.4	0.16~0.47(1); <0.094~4.50 (17)
Adrenal Axis Hormone		
Adrenocorticotropic hormone (ACTH) [pmol/L]	3.23	<50pg/ml=11.01pmol/l (17)
Cortisol [μg/dL]	8.30	10.14~19.93 (17)
Dehydroepiandrosterone sulfate (DHEAs) [µmol/L]	1.54	1.83±1.38 (17)
Androstenedione [nmol/L]	2.98	2.40±1.85 (17)
17-Hydroxyprogesterone (17OHP) [ng/ml]	1.10	0.099~1.55 (17)
GnRHa(triptorelin) Stimulation Test		
60min FSH [IU/L]	7.79	60min LH≥ 5.0 and 60min LH/FSH ratio ≥ 0.6 indicates HPG axis activation (18).
60min LH [IU/L]	10.77	

Given the biochemical profile, hCG-secreting intracranial non-germinomatous germ cell tumor (NGGCT) was suspected. The patient received four chemotherapy cycles of cisplatin (DDP)/etoposide (VP16) alternating with ifosfamide (IFO)/etoposide (VP16), with a three-week interval between each cycle (10). After two cycles of chemotherapy, there was no significant response in blood/cerebrospinal fluid β-HCG levels. Therefore, GnRHa (monthly release triptorelin) treatment was initiated for CPP at the start of the third cycle. However, after four chemotherapy cycles, β-hCG levels in both serum and CSF remained elevated, with no change in cranial imaging. She subsequently underwent pineal radiotherapy and cyst resection at a specialized neurosurgical hospital. Histopathology revealed a central neurocytoma (small round cell tumor with synaptophysin expression), and hCG concentrations showed no decline postoperatively (**Table 2**).

**Table 2:** Comparison of β-hCG assays in a familial hCG syndrome pedigree.

Time point	Age (years)	Serum β-hCG (IU/L)		CSF β-hCG (IU/L)	
		(Abbott/ total βhCG)	(Roche/ hCG+β)	(Abbott/ total βhCG)	(Roche/ hCG+β)
Proband At admission	8.73	45.58	44.0 (147.4&)	103.22	ND (241.9&)
	8.76	40.33			
After 1st chemotherapy cycle	8.79	47.28	44.0		
After 2nd chemotherapy cycle	8.83	35.72		104.9	
After 3rd chemotherapy cycle	8.88	39.82		101.6	
After 4th chemotherapy cycle	8.95	39.17		109.7	
Pre-radiotherapy*	8.99		130.0#		260.0#
			99.2#		
Post-pineal radiotherapy*	9.02		110.1#		
Pre-resection*	9.05		102.8#		
Post-pineal cyst resection*	9.05		117.5#		
Follow-up					
4 months post-op	9.38	63.43			
15 months post-op	10.35	40.95			
22 months post-op	10.91	43.47			
28 months post-op	11.41	39.04			
35 months post-op	12.01	37.85			
Younger brother (TV 3ml)	6	64.58	ND	ND	
Elder brother (TV 10ml)	10.75	<1.2	ND	ND	
Mother Day 14 of the menstrual cycle Day 20 in another menstrual cycle	31	43.79 79.94	ND ND	ND ND	
Father	36	<1.2	ND	ND	
Maternal grandmother**	60	<1.2	ND	ND	

&Roche auto-dilution up to 15-fold; *brain hospital; **local hospital; #Diluted; exact dilution factor unspecified; TV, testicular volume; ND, not determined.

**Figure 3:**
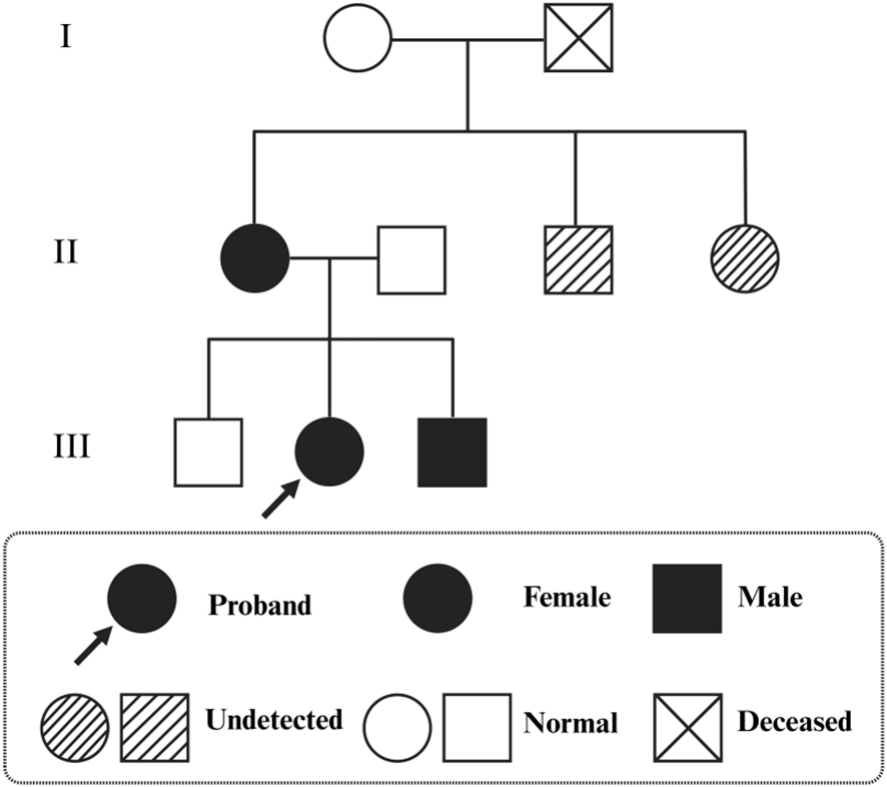
Pedigree of the family. Squares represent males and circles represent females; a diagonal line indicates deceased individuals. Filled symbols denote affected individuals, and open symbols denote unaffected individuals. Half-shaded symbols indicate carriers. The proband is indicated by an arrow.

Due to persistently elevated β-hCG levels and lack of therapeutic response, the patient was referred back to our hospital. The patient’s parents were non-consanguineous, and both of her brothers were healthy. Subsequent family screening revealed elevated β-hCG in the mother and younger brother (**Table 2**; **Figure 3**). The mother’s urine pregnancy test was negative, excluding pregnancy. Whole-exome sequencing in the proband did not identify pathogenic variants.

Based on these findings, a diagnosis of familial hCG syndrome was established. Additionally, the onset and progression of precocious puberty are central in nature and unrelated to the elevated hCG levels, indicating hCG-independent central precocious puberty. The patient continued GnRH agonist therapy for CPP, resulting in improved predicted adult height.

## 4 Follow-up

During 43 months of follow-up, serum β-hCG remained persistently elevated but fluctuated between 37.85-63.43 IU/L (Abbott Architect), while clinical condition remained stable.

## 5 Discussion

Familial hCG syndrome was first identified in 2004 (2). A woman with preoperative elevation of hCG—treated empirically with chemotherapy for presumed choriocarcinoma after pregnancy was excluded—showed no decline in hCG. She was referred to the U.S. hCG Reference Service, where, after excluding other causes (including pituitary hCG) and negative imaging, both her sister and mother were incidentally found to have similarly elevated hCG, suggesting a hereditary basis. The term familial hCG syndrome was introduced in 2008. To date, approximately 14 kindreds and one isolated case have been reported globally, comprising 15 probands (age 22–58 years; 6 men, 9 women) and 23 relatives (age 8 months–56 years; 12 men, 11 women) (2–8). While the majority of previously reported probands were asymptomatic individuals identified incidentally during routine screenings, the case presented here exhibits a unique and deceptive phenotype: a pediatric patient presenting with central precocious puberty who was found to have elevated hCG not only in the serum but, significantly, in the CSF at levels exceeding those in the blood. This represents the first global report of familial hCG syndrome accompanied by elevated CSF hCG.

The diagnostic trajectory of this case highlights a critical conflict between established clinical guidelines and rare genetic anomalies. Given a subset of precocious puberty is tumor-associated and hCG serves as a tumor marker outside pregnancy, the 2011 Chinese guideline on precocious puberty includes hCG (and AFP) in the basic initial screening panel (12). Although the patient’s basal and GnRH-stimulated gonadotropin profiles indicated a GnRH-dependent (central) process, the presence of a pineal cyst on MRI combined with markedly elevated CSF hCG inevitably led to a provisional diagnosis of malignancy. This biochemical profile mimics intracranial germ cell tumors, where elevated CSF hCG is typically considered a sensitive marker for intrathecal tumor secretion. Consequently, our patient underwent unnecessary chemotherapy, radiotherapy, and surgery. And the patient was treated with a GnRH agonist to suppress the hypothalamic-pituitary-gonadal axis, slow bone-age advancement and preserve adult height. The diagnosis was only corrected after the lack of therapeutic response prompted segregation analysis, which revealed elevated serum hCG in her asymptomatic mother and brother. Throughout treatment, serum and CSF hCG levels fluctuated independently of gonadotropin suppression, supporting our conclusion that the hCG elevation was incidental and related to familial hCG syndrome rather than a driver of precocious puberty.

A Delphi consensus allows treatment without biopsy when imaging suggests intracranial GCT and markers are elevated (13). Although our patient’s imaging showed a pineal cyst rather than a classic GCT, the high CSF hCG prompted NGGCT-localized management. No biochemical response followed four chemotherapy cycles, pineal radiotherapy, or surgery, pointing to benign hCG elevation (10). Elevated serum hCG in the mother (regular cycles; days 14 and 19) and in a prepubertal brother (normal FSH/LH/T) confirmed familial hCG syndrome; negative WES is expected given the absence of a known causal gene.

Multiple studies have indicated that measuring hCG in cerebrospinal fluid (CSF) and serum provides important clues for diagnosing intracranial germ cell tumors (iGCTs) (13–15). The 2015 international consensus on iGCT management and the 2022 Japanese guideline both recommend assaying CSF hCG and α-fetoprotein (AFP) (13, 14). The rationale is that, in tumors within the craniospinal axis that contain trophoblastic elements—whether primary or metastatic—trophoblastic cells secrete hCG that first diffuses into the CSF and only subsequently crosses the blood–brain barrier into the circulation. Consequently, CSF hCG rises earlier than serum hCG and is typically higher, making CSF-hCG a more sensitive marker than serum hCG for detecting intrathecal hCG secretion (15).

To explain the unprecedented CSF elevation in this benign condition, we hypothesize that a genetic alteration may drive expression and secretion, within certain intracranial cells, of hCG species characterized by high degradability and/or impaired recognition of the C-terminal peptide (CTP). If the secretory pathway communicates with the subarachnoid space, hCG could enter the CSF directly, yielding a biochemical profile dominated by marked intracranial hCG elevation, analogous to that seen in intracranial germ cell tumors.

This study has notable strengths and limitations. The primary strength lies in the comprehensive longitudinal data, including paired serum/CSF monitoring and pathological confirmation (resection of the pineal cyst) that definitively excluded malignancy. Furthermore, the rigorous family screening provided the gold standard for diagnosis. However, limitations must be acknowledged. First, the lack of the Siemens Immulite assay prevented us from confirming the specific presence of CTP-deleted variants. Second, Whole Exome Sequencing (WES) was negative; this is not uncommon in familial hCG syndrome, as the specific genetic drivers often involve complex structural variations in the *chorionic gonadotropin beta* (*CGB*) gene cluster that are difficult to resolve with standard WES. Finally, our hypothesis regarding the intracranial cellular source of hCG remains speculative without tissue-specific expression studies. Furthermore, we must acknowledge a significant clinical limitation in the management of this case: the initiation of empirical oncological treatment without prior histological confirmation. While international consensus guidelines sometimes permit treating intracranial GCTs based on pathognomonic marker elevation without biopsy, the radiological ambiguity presented by our patient—a purely cystic pineal lesion rather than a typical solid or mixed tumor—should have prompted greater caution. In retrospect, treating the patient based solely on biochemical findings in the face of atypical imaging carried substantial risks. A diagnostic biopsy should have been more strongly considered before initiating chemotherapy and radiotherapy. Before subjecting a pediatric patient to the physical and psychological burdens of oncology treatments, a simple and non-invasive family screening for hCG should be prioritized as a critical exclusionary step.

Despite these limitations, the clinical lessons are profound. Familial hCG syndrome must be approached as a diagnosis of exclusion, particularly when there is a “mismatch” between biochemical severity and imaging findings (e.g., high CSF hCG but no visible tumor on MRI). Discordance between assay platforms (Roche vs. Abbott) can further serve as a diagnostic clue. In conclusion, when concurrent elevation of hCG is detected in blood and CSF—even when CSF levels exceed those in the peripheral blood—it should not immediately trigger treatment for an intracranial tumor. Instead, clinical decision-making must be integrated with imaging findings. If imaging does not suggest a tumor, or if the response to empirical anti-tumor therapy regarding a suspicious lesion is unsatisfactory, the possibility of familial hCG syndrome should be considered. In such scenarios, it is essential to employ multiple assay methods to evaluate hCG levels and, crucially, to conduct comprehensive hCG screening of family members.

## Data Availability

The original contributions presented in the study are included in the article/supplementary material. Further inquiries can be directed to the corresponding authors.
